# An update on immunopathogenesis, diagnosis, and treatment of multiple sclerosis

**DOI:** 10.1002/brb3.362

**Published:** 2015-08-03

**Authors:** Neeta Garg, Thomas W Smith

**Affiliations:** 1 Department of Neurology, University of Massachusetts Medical SchoolWorcester, Massachusetts, 01655; 2 Department of Pathology, University of Massachusetts Medical SchoolWorcester, Massachusetts, 01655

**Keywords:** Demyelination, diagnosis, etiology, immunomodulator, multiple sclerosis, pathogenesis, treatment

## Abstract

**Background:**

Multiple sclerosis is an acquired demyelinating disease of the central nervous system. It is the second most common cause of disability in adults in United States after head trauma.

**Discussion:**

The etiology of MS is probably multifactorial, related to genetic, environmental, and several other factors. The pathogenesis is not fully understood but is believed to involve T-cell-mediated inflammation directed against myelin and other related proteins with a possible role for B cells. The McDonald criteria have been proposed and revised over the years to guide the diagnosis of MS and are based on clinical presentation and magnetic resonance imaging (MRI) of the brain and spinal cord to establish dissemination in time and space. The treatment of MS includes disease modification with immunomodulator drugs and symptom management to address the specific symptoms such as fatigue, spasticity, and pain.

**Conclusion:**

An update on etiology, pathogenesis, diagnosis, and immunomodulatory treatment of MS is presented.

## Introduction

Multiple sclerosis is an autoimmune inflammatory disorder of CNS of unknown etiology, characterized by demyelination and variable degrees of axonal loss. The disease affects mostly young women (between ages 20 and 40 years) and is one of leading causes of disability in young adults in United States (Orton et al. 2006; Kister et al. 2013). Its prevalence in the United States is about 400,000 and over two million people are affected worldwide with an expected increase in the number of cases in future (Weinshenker 1996; Mayr et al. 2003). The disease appears to be more common in northern hemisphere and there is some genetic susceptibility as well in individuals of Scandinavian or northern European ancestry (Williams et al. 1995; Compston and Coles 2008). The etiology although unknown presumably involves interaction between genetic, environmental, and other factors triggering an aberrant autoimmune attack resulting in damage to myelin and axons (Bruck and Stadelmann 2005). The course of MS can be variable with a significant proportion of patients experiencing some progression following the initial relapsing remitting phase leading to significant disability (Weinshenker et al. 1989; Lublin and Reingold 1996). Much progress has been made in the past two decades in treating MS with the advent of effective immunomodulatory therapies which can potentially slow down the progression and alter the disease course.

## Etiology

The etiology of MS remains unknown; however, it is believed to be caused by immune dysregulation triggered by genetic and environmental factors (Ascherio and Munger 2007a,b). Although MS is not an inherited disease, there is a strong genetic component to its etiology as evidenced by clustering of MS cases within families. The risk of MS among first-degree relatives of MS patients is 10–50 times higher than the general population (absolute risk 2–5%); the concordance rate in monzygotic twins is about one-third (Weinshenker 1996; Kantarci 2008). Linkage analysis studies have revealed several gene loci as risk factors, with the major histocompatibility complex (MHC) HLA DR15/DQ6 allele being the strongest one (Barcellos et al. 2003; Sawcer et al. 2011). More recently, alleles of interleukin-2 receptor alpha gene (IL2RA) and interleukin-7 receptor alpha gene (IL7RA) have also been identified as inheritable risk factors (Hafler et al. 2007; Sawcer et al. 2011). However, most of the genetic factors underlying susceptibility still remain to be defined. Furthermore, genetic susceptibility does not fully explain the changes in MS risk that occur with migration suggesting a likely role for environmental factors.

Among environmental factors, Epstein-Barr virus (EBV) infection and vitamin D deficiency have been extensively studied and strongly linked to MS risk (Ascherio and Munger 2007a,b) MS is prevalent in geographic areas farther away from the equator (Simpson et al. 2011). Low vitamin D levels from reduced sun exposure may be a factor contributing to increased susceptibility to MS in these regions (Ascherio and Munger 2007b; Correale et al. 2009; Ascherio et al. 2010). Studies have suggested that higher levels of vitamin D have a possible protective role in certain susceptible patient populations (Munger et al. 2004, 2006). The risk of developing MS is approximately 15-fold higher among individuals with a history of EBV infection in childhood and about 30-fold higher among those infected with EBV in adolescence or later in life (Ascherio 2013). However, the difference in the risk of MS among migrants from high to low MS prevalence areas suggests that other infectious or noninfectious factors in addition to EBV may be involved (Ascherio and Munger 2007a,b). The “hygiene hypothesis,” supported by many epidemiological observations, suggests that improved sanitation and reduced childhood infections in developed countries may account for the increased rates of autoimmune diseases (T-helper 1 mediated) and allergy (T-helper 2 mediated) (Conradi et al. 2011). However, this hypothesis does not explain the higher MS prevalence in rural compared to urban areas (with expected improved sanitation) reported in some studies (Sotgiu et al. 2003).

Cigarette smoking has also been proposed at a potential environmental risk factor with several studies reporting an association between smoking and MS risk and disease activity (Wingerchuk 2012). The odds ratio for developing MS is approximately 1.5 for smokers compared with nonsmokers (Wingerchuk 2012; Fragoso 2014). As with other risk factors, smoking appears to influence the MS susceptibility in conjunction with the genetic and other environmental factors.

There is no specific diet associated with increased risk of MS. The role of dietary factors appears to be complex and related to the influence of multiple dietary components including vitamin A and D, salt, omega-3-unsaturated fatty acid, and polyphenol on immune regulation. Some recent reports have suggested that salt modulates the differentiation of human and mouse Th17 cells (Kleinewietfeld et al. 2013; Wu et al. 2013). A more aggressive course of experimental autoimmune encephalomyelitis (EAE) was observed in mice fed a high sodium diet. In a small observational study, higher sodium intake was associated with increased clinical and radiological disease activity in patients with MS (Farez et al. 2015).

The potential risk factors for MS are listed in Table .

**Table 1:** Potential risk factors for multiple sclerosis

Female gender
Caucasian race
Genetic
HLA DR15/DQ6, IL2RA and IL7RA alleles
Infections
Epstein–Barr virus (EBV) infection
Temperate climate
Low vitamin D level
Lack of sunlight exposure
Cigarette smoking

## Immunopathogenes

The pathogenesis of MS involves immune attack against CNS antigens mediated through activated CD4+ myelin-reactive T cells with a possible contribution by B cells. Much of our understanding of immunopathogenesis of MS is derived from the study of experimental autoimmune encephalomyelitis (EAE), an animal model of CNS inflammatory demyelination that can be induced by peripheral immunization with myelin protein components. EAE shares many of the histologic features of MS including active demyelination, oligodendrocyte and axonal loss, all of which are presumably mediated by myelin specific T cells (Yong 2004; Gold et al. 2006). The immunopathogenesis of MS is thought to involve a breach of self-tolerance toward myelin and other CNS antigens resulting in persistent peripheral activation of autoreactive T cells (Hafler et al. 2005; Selter and Hemmer 2013). In a genetically susceptible individual, this loss of self-tolerance may be triggered by an environmental antigen, presumably an infectious agent such as a virus. The infection could cause bystander activation of T cells or result in release of autoantigens due to cellular damage, which can then lead to activation of T cells by cross reactivity between an endogenous protein (e.g., myelin basic protein) and the pathogenic exogenous protein (viral or bacterial antigen), a process known as molecular mimicry (Fujinami and Oldstone 1985; Wucherpfennig and Strominger 1995; Aichele et al. 1996; Gran et al. 1999; O’Connor et al. 2001).

**Figure 1:**
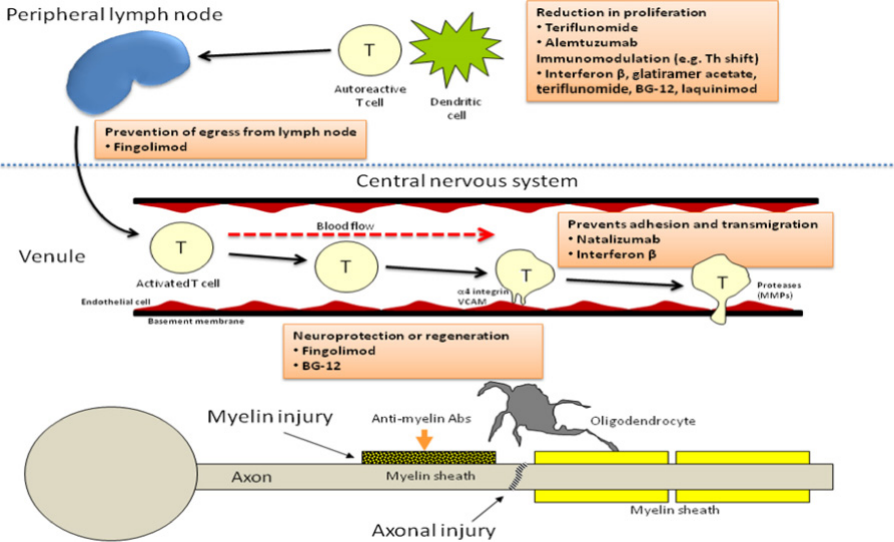
Immunopathogenic mechanisms in MS and proposed targets of different disease modifying therapies.

As depicted in Figure1, once activated in the periphery, myelin-reactive T cells are able to migrate across the blood–brain barrier (BBB). The transmigration process involves interaction between very late antigen-4 (VLA-4) present on T lymphocytes and the vascular cell adhesion molecule-1 (VCAM-1) expressed on capillary endothelial cells; this process is facilitated by expression and upregulation of various adhesion molecules, chemokines, and matrix metalloproteinases (MMPs) (Yong 2004; Gold and Wolinsky 2011). After entering the CNS, the autoreactive peripherally activated T cells can be reactivated upon encountering the autoantigenic peptides within the brain parenchyma in the context of MHC class II molecules expressed by local antigen presenting cells (dendritic cells, macrophages, and B cells) triggering an inflammatory cascade leading to release of cytokines and chemokines, recruitment of additional inflammatory cells including T cells, monocytes, and B cells and persistent activation of microglia and macrophages resulting in myelin damage (Hemmer et al. 2002; Frohman et al. 2006; Inglese 2006). The local inflammation and demyelination results in exposure of sequestered myelin autoantigens providing an additional target for self-reactive T cells, a phenomenon called “epitope spreading” (Miller et al. 1997). Activation of resident CNS glial cells (such as microglia) results in persistent inflammation even in absence of further infiltration of exogenous inflammatory cells (O’Connor et al. 2001). The evidence based on animal studies suggests that CD4^+^ T-helper 1 (T_H_1) cells which release proinflammatory cytokines such as interferon-gamma, interleukin-2 (IL-2), and tumor necrosis factor-*α* (TNF-*α*) are the key players in mediating inflammation in MS with some role for the novel CD4^+^ T-helper-17 (T_H_17) cell subset which secretes IL-17 (O’Connor et al. 2001; Selter and Hemmer 2013). The CD4^+^ T-helper 2 (T_H_2) cells, which secret interleukins 4, 5, and 10 are believed to have a counter regulatory role limiting the T_H_1-cell-mediated injury (Tzartos et al. 2008). The T_H_1/T_H_2 paradigm is more apparent in EAE; in MS, indirect evidence exists for a predominant role of Th1 cells based on the success of therapies that shift the cytokine profile away from Th1 toward Th2. CD8+ T cells are believed to be involved as well and can induce axonal pathology by direct injury to MHC I/antigen expressing cells such as neurons and oligodendrocytes (Batoulis et al. 2010). The contribution of B cells to MS pathogenesis (possibly through autoantibody secretion and antigen presentation to T cells) has recently been recognized and is supported by observed pathologic heterogeneity of MS lesions, the presence of meningeal inflammation and B-cell follicle-like structures adjacent to subpial cortical lesions, and the success of B-cell-based immunotherapies (O’Connor et al. 2001; Batoulis et al. 2010; Naismith et al. 2010).

Although demyelination is the hallmark of MS pathology, early axonal injury and axonal loss also occur and may drive disability progression (Trapp et al. 1998). The exact mechanism(s) of both myelin and axonal injury are not completely understood, but are likely to include both direct injury to myelin and oligodendrocytes, and axons by CD4+ and CD8+ T lymphocytes, activated microglia/macrophages, and/or antibody and complement as well as the indirect effects of proinflammatory cytokines such as IL-1beta, TNF-*α*, nitric oxide, and MMPs (Lucchinetti et al. 2000; Hemmer et al. 2002; Gold and Wolinsky 2011). Meningeal inflammatory infiltrates reported in association with subpial cortical lesions may contribute to cortical inflammation and disability in some cases (Howell et al. 2011; Lucchinetti et al. 2011).

## Pathology

The MS plaques or lesions are focal areas of demyelination associated with variable inflammation and axonal loss that predominantly affect the white matter of the brain, spinal cord, and optic nerves but can also involve the cerebral cortex including subpial regions (Sobel and Moore 2008; Popescu and Lucchinetti 2012). The inflammatory infiltrates associated with plaques consist of activated T cells (predominantly CD8+ with variable presence of CD4+ cells), activated macrophages/microglia, plasma cells, and B cells (Hauser et al. 1986; O’Connor et al. 2001). MS plaques can be further classified histologically as active, chronic, and remyelinated. Active lesions are common in relapsing remitting MS and are characterized by myelin degradation (with relative axonal preservation), macrophage infiltration, reactive astrocytes, and perivascular and parenchymal inflammation (Bruck et al. 1995; Frischer et al. 2009). Chronic or inactive plaques are more often seen in patients with progressive disease and are associated with more extensive demyelination, often with marked axonal depletion, loss of oligodendrocytes, and relative absence of active inflammation (Prineas et al. 2001; Sobel and Moore 2008; Popescu and Lucchinetti 2012). Remyelinated plaques are seen within or more often at the margins of active plaques and contain thinly myelinated axons and often increased numbers of oligodendrocyte precursor cells (Bruck et al. 1995; Popescu and Lucchinetti 2012). “Shadow plaques” are lesions that show more diffuse (but still incomplete) remyelination and are seen in patients with relapsing and progressive disease (Barkhof et al. 2003). The presence of cortical demyelination and axonal loss has been increasingly recognized in early MS (Trapp et al. 1998; Cifelli et al. 2002). Lucchinetti and colleagues have described four distinct immmopathological patterns (pattern I with macrophage and T-cell predominance, II with additional immunoglobulin and complement deposition, III with apoptotic oligodendrocyte loss, and the rare type IV pattern with nonapoptotic death of oligodendrocytes) in active MS lesions, suggesting that there may be pathological heterogeneity among MS patients (Lucchinetti et al. 2000). However, the observed pathologic heterogeneity may not be exclusive to a subset of MS patients and is probably related to the stage of disease in a given patient (Barnett and Prineas 2004). Cortical involvement can occur in MS and may reflect either the presence of cortical demyelination or actual neuronal loss. Within the cortex, three distinct lesion types have been described based on the location of the plaques: subpial, intracortical, and leukocortical (Bogdan et al. 2013). Cortical lesions seen in early MS are usually highly inflammatory and correlate with cognitive impairment (Geurts and Barkhof 2008; Lucchinetti et al. 2011).

## Clinical Presentation and Diagnosis

The clinical symptoms and signs of MS are variable and may result from involvement of sensory, motor, visual, and brainstem pathways. The majority of patients with MS initially present with relapsing remitting episodes of new or recurrent neurological symptoms. The first clinical event in these patients, termed clinically isolated syndrome (CIS), can be optic neuritis, incomplete myelitis, or brainstem syndrome (Miller et al. 2005). The presence of classic demyelination lesions on baseline brain or spinal cord MRI is the most important predictor of having a second relapse in CIS patients (Filippi et al. 1994). The presence of cerebrospinal fluid (CSF) abnormalities (positive oligoclonal bands) may have additional prognostic value in patients with CIS and positive brain MRI (Miller et al. 2005; Awad et al. 2010). A variable proportion of patients with relapsing remitting MS (25–40%) develop secondary progressive disease over time with progressive accumulation of disability with infrequent or no relapses (Lublin and Reingold 1996). Primary progressive MS (seen in approximately 10–15% patients) is defined by progressive accumulation of disability from the onset with no or minor relapses and typically presents with a progressive myelopathy with an older age of onset and involving a higher proportion of men (Miller and Leary 2007). Both primary and secondary progressive MS share some clinical and imaging features and are now considered to be part of the progressive disease spectrum (Lublin and Reingold 1996; Ingle et al. 2005; Lublin et al. 2014). The progressive relapsing form of MS with worsening disability from onset and clear acute relapses with or without full recovery is now considered to be progressive disease with disease activity (Lublin et al. 2014).

There is no single diagnostic test for MS and the diagnosis is usually based on the clinical presentation, supported by neuroimaging and in some cases by CSF analysis (to look for inflammatory markers oligoclonal bands and/or elevated IgG index) and evoked potential studies (to look for clinically silent lesion in visual, brainstem, or spinal cord pathways). CSF inflammatory markers are present in up to 85% patients with MS (Link and Huang 2006); IgG index is less sensitive and specific than oligoclonal bands Awad et al. 2010). There have been several proposed diagnostic criteria incorporating the clinical and ancillary data, the most commonly used one is the McDonald criteria initially proposed in 2001 and revised in 2005 and most recently in 2011 (Polman et al. 2011). The basic concept behind these criteria is demonstration of dissemination in time (DIT) and space using the clinical and/or MRI data. A detailed discussion of McDonald criteria is beyond the scope of this review; in summary, the definitive diagnosis of MS requires ≥2 attacks or objective clinical evidence of ≥ 2 lesions or objective clinical evidence of 1 lesion with historical evidence of a prior attack. With one clinical attack, DIT can be demonstrated by presence of asymptomatic gadolinium-enhancing and nonenhancing lesions at any time or by presence of new lesions on a follow-up scan obtained anytime after the initial symptom onset or the simultaneous (see Table 1996). Dissemination in space (DIS) in a patient with two clinical attacks but objective evidence of one lesion can be demonstrated by using the MRI criteria detailed in Table 2013. The criteria for primary progressive MS include 1 year of disease progression plus two of the following criteria: a. evidence of DIS in brain, b. DIS in spinal cord (≥ 2 T2 lesions in the cord), c. positive CSF oligoclonal bands and/or elevated IgG index.

**Table 2:** McDonald MRI criteria for demonstration of DIT (Polman et al. 2011)

DIT Can be Demonstrated by
1. A new T2 and/or gadolinium-enhancing lesion(s) on follow-up MRI, with reference to a baseline scan, irrespective of the timing of the baseline MRI
2. Simultaneous presence of asymptomatic gadolinium-enhancing and nonenhancing lesions at any time

MRI, magnetic resonance imaging; DIT, lesion dissemination in time.

Based on Montalban et al. 2010.

Adapted from Polman et al. (2011).

**Table 3:** McDonald MRI criteria for demonstration of DIS (Polman et al. 2011)

DIS can be demonstrated by ≥ 1 T2 lesions^1^ in at least 2 of the 4 area of the CNS
Periventricular
Juxtacortical
Infratentorial
Spinal cord^2^

MRI, magnetic resonance imaging; DIS, lesion dissemination in space; CNS, central nervous system.

Based on Swanton et al. 2006, 2007.

Adapted from Polman et al. (2011).

1 Gadolinium enhancement of lesions is not required for DIS.

2 If a subject has a brainstem or spinal cord syndrome, the symptomatic lesions are excluded from the Criteria and do not contribute to lesion count.

**Figure 2:**
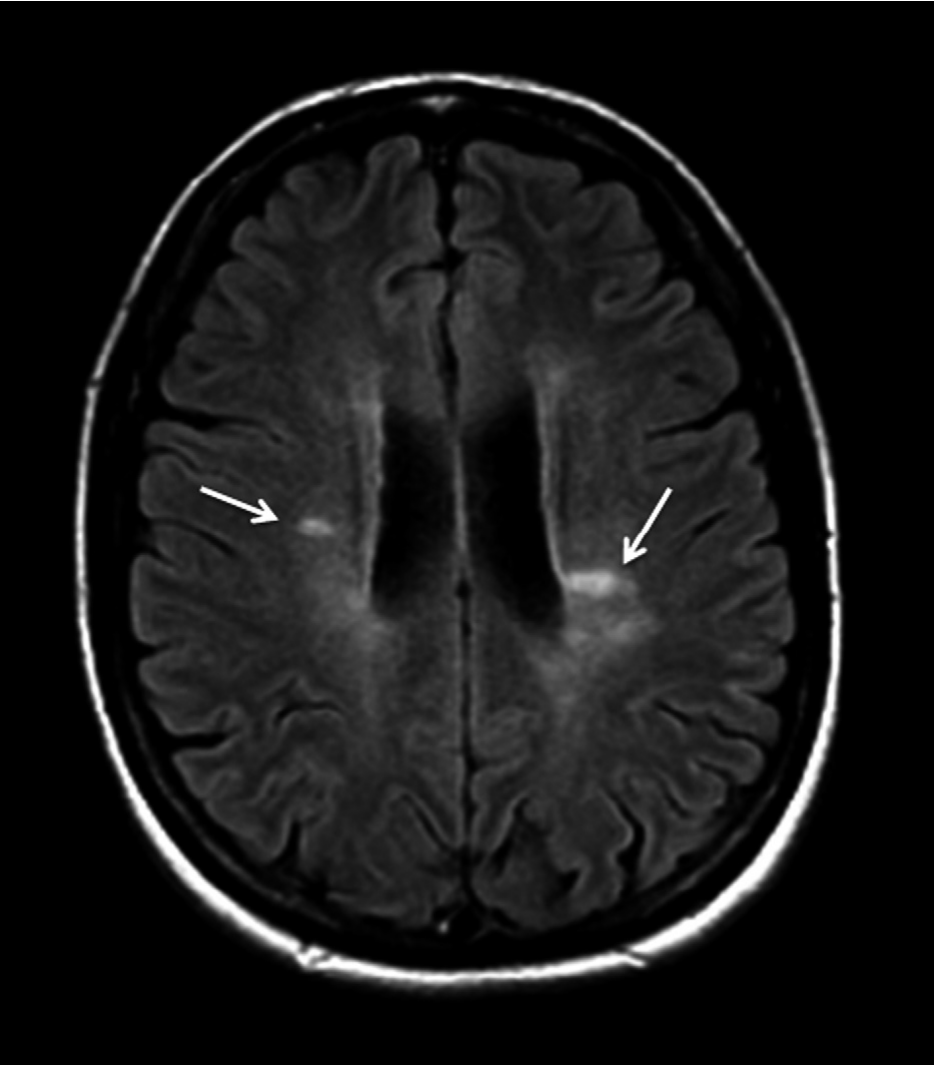
Brain MRI axial fluid attenuated inversion recovery (FLAIR) image shows the characteristic periventricular areas of increased signal intensity (arrows) that are oriented perpendicular to and often contiguous with the lateral ventricles.

In patients presenting with typical relapsing remitting symptoms and classic demyelination lesions (e.g., shown in Fig.2) on neuroimaging meeting the radiological criteria, the differential diagnosis is limited and often no further diagnostic testing is indicated in these cases.

The differential diagnosis in other cases depends on the clinical presentation and is outlined in Table 2007a.

**Table 4:** Differential diagnosis of multiple sclerosis

Optic neuritis/neuropathy
Inflammatory, neuromyelitis optica (NMO) spectrum disorder, genetic, ischemic
Myelitis/myelopathy—
Inflammatory demyelination—idiopathic, postviral, postvaccinialNMO spectrum disorder, Autoimmune–systemic lupus erythematosus, antiphospholipid antibody syndrome, other systemic autoimmune disorders
Infectious (Lyme disease, HIV, viral, others)
Ischemic/vascular
Others–compressive, nutritional
Brainstem syndrome
Stroke, tumor, vasculitis (lupus, Sjögren’s syndrome, Behçet’s disease)
Cerebral white matter lesions
Small vessel disease (Leukoaraiosis)
Migraine
Primary CNS vasculitis
Sarcoidosis
CADASIL (Cerebral Autosomal Dominant Arteriopathy with Subcortical Infarcts and Leukoencephalopathy)

### Atypical presentation or variants of MS

There are some less common clinical variants of MS which present with atypical clinical and radiological features, these include tumefactive MS, Balo’s concentric sclerosis, and Marburg disease. The radiological hallmark of tumefactive MS is a large solitary >2 cm lesion associated with mass effect, edema and/or ring enhancement (hence the name tumefactive). The clinical symptoms depend on the size and location of the lesion and often include aphasia, agnosia, seizures and visual field defects, not typically seen in CIS or RRMS patients (Lucchinetti et al. 2008). Marburg’s disease and Balo’s concentric sclerosis are characterized by a rapidly evolving fulminant clinical course and poor prognosis. The Marburg variant has the distinct radiological feature of large tumor-like multifocal demyelinating lesions in deep white matter; the pathological changes are similar to those of classicMS but may appear more destructive and have more inflammatory infiltrates (Karussis 2014). The pathological changes seen in Balo’s concentric sclerosis are quite unique and consist of alternating bands of normally myelinated or remyelinated, and demyelinated white matter; this pattern has been described as resembling hypoxia-induced injury (Stadelmann et al. 2005). The MRI may show alternating isointense and hypointense concentric rings with partial enhancement on T1-weighted images (Zettl et al. 2012; Karussis 2014).

## Therapeutic Options

The management of MS includes treatment with immunomodulatory agents that help alter the course of the disease, symptomatic management focusing on relieving specific symptoms such as fatigue, spasticity, bladder dysfunction, and pain (not discussed in this review). Corticosteroids (methylprednisolone) and adrenocorticotropic hormone (ACTH) have anti-inflammatory and immunomodulatory effects and are typically used to treat acute relapse to hasten the recovery (Berkovich 2013). The immunomodulatory therapies (IMT) used in long-term disease modification are discussed in the next section.

## Immunomodulatory Therapies

The most significant progress in the treatment of MS in the last two decades has been the development of IMT. Since the introduction of first immunomodulating medication, interferon beta-1b in 1993, several other medications with different mechanism of action (figure1), mode and frequency of administration have become available. Currently, there are 12 medications approved for treatment of MS, including six self-injectable, three infusion based, and three oral medications as listed in Table 2007b.

**Table 5:** Approved therapies for multiple sclerosis

Medication	Dose	Route	Frequency	Major side effects
First-line therapies				
Beta-interferon-1b (Betaseron®, Bayer HealthCare Pharmaceuticals Inc., Whippany, NJ)	250 µg	SC	Every other day	Flu-like symptoms, injection site reactions, liver enzyme elevation, thyroid abnormalities, leukopenia or anemia, and depression
Beta-interferon-1a (Avonex®, Biogen Idec, Cambridge, MA )	30 µg	IM	Once a week	
Peginterferon beta-1a (Plegridy®, Biogen Idec, Cambridge, MA)	125 µg	SC	Every 14 days	
Beta-interferon-1a (Rebif®, EMD Serono, Inc. Rockland, MA )	44 µg	SC	Three times weekly	
Beta-interferon-1b (Extavia®, Bayer HealthCare Pharmaceuticals Inc., Montville, NJ)	250 µg	SC	Every other day	
Glatiramer acetate (Copaxone®, TEVA Neuroscience, Inc., Overland Park, KS )	20 mg	SC	Daily	Local injection site reactions, postinjection reaction (flushing, chest tightness, palpitation, and dyspnea) and rare lipoatrophy with prolonged use
	40 mg	SC	Three times weekly	
Second line therapies				
Natalizumab (Tysabri®, Biogen Idec, Cambridge, MA)	300 mg	IV	Every 4 weeks	Hepatotoxicity, infusion reactions, progressive multifocal encephalopathy (PML)
Mitoxantrone (Novantrone®, EMD Serono, Inc. Rockland, MA)	Weight-based dose	IV	Every 3 months	Cardiotoxicity, secondary leukemia
Fingolimod (Gilenya®, Novartis Pharma Stein AG Stein, Switzerland)	0.5 mg	Oral	Once daily	First dose bradycardia, atrioventricular block, herpes virus infection, macular edema, elevated blood pressure, rare risk of PML
Teriflunomide (Aubagio®, Genzyme Corporation, Cambridge, MA)	7 and 14 mg	Oral	Once daily	Hair loss, headache, diarrhea, hepatotoxicity, teratogenicity, increased risk of infections due to lymphopenia
Dimethyl fumarate (Tecfidera®, Biogen Idec., Cambridge, MA )	240 mg	Oral	Twice daily	GI effects-nausea, diarrhea, abdominal pain, flushing, pruritus, liver enzyme elevation, lymphopenia, rare cases of PML
Alemtuzumab (Lemtrada®, Genzyme Corporation, Cambridge, MA )	12 mg or 24 mg	IV	Per day (5 days in year 1, 3 days in year 2)	Serious infusion reactions, secondary autoimmune diseases-thryoiditis, thrombocytopenia, anti-glomerual basement membrane disease, increased risk of malignancies—thyroid cancer, melanoma, lymphoproliferative disorders

The mechanism of action of IMT used for treatment of MS is broad suppression of the immune response mediated by autoreactive lymphocytes; most of these are effective in relapsing remitting MS where inflammatory demyelination is the primary process (Weinstock-Guttman et al. 1995; Rudick et al. 1997; Damal et al. 2013). The goal of these therapies is to reduce the frequency of relapses and number of MRI lesions (new, enlarging and/or enhancing T2 lesions), and slow the disability progression. Most of these agents have shown good efficacy in patients with relapsing remitting MS and clinically isolated syndrome, however, their benefit in patients with progressive disease has been questionable (Filippini et al. 2013). The mechanism of action and side-effect profile of different IMTs are briefly discussed here with the exception of alemtuzumab, the latest medication to be approved for treatment of MS.

## Beta-Interferon

Interferons (IFNs) are endogenous proteins that are involved in immune response against viral and bacterial agents and were the first class of disease modifying agents developed for treatment of MS. The beta-interferons (IFN-*β*) have multiple actions including stabilizing the BBB thereby limiting the entry of T cells into the CNS, modulating T- and B-cell function, and altering the expression of cytokines (Yong et al. 1998; Weber et al. 1999; Dhib-Jalbut 2002). Several different preparations of IFN- *β* are available and are listed in Table 2007b. Both IFN- *β*1a and IFN-*β*1b have shown similar efficacy and are considered first line agents for treating patients with relapsing MS and CIS (Rudick et al. 1997). Although two different trials with IFN- *β*1b showed conflicting results in secondary progressive MS, it may be indicated in patients with continued relapses (Kappos et al. 2004). The side effects of beta-interferon include flu-like symptoms, depression, liver enzyme elevation, thyroid abnormalities, leukopenia or anemia, and injection site reactions (Rudick et al. 1997).

## Glatiramer Acetate

Glatiramer acetate (GA) or Copolymer 1 is a synthetic complex of four amino acids that mimics myelin basic protein (MBP), one of the autoantigens targeted by the T cells. Due to its structural similarity to MBP, GA blocks the formation of myelin reactive T cells and induces GA-specific regulatory T-cell expression and Th2 anti-inflammatory cytokine production (bystander suppression) (Wolinsky 1995; Rudick et al. 1997; Gran et al. 2000; Dhib-Jalbut 2002; Ruggieri et al. 2007). The clinical efficacy of GA in terms of reducing relapse rate and MRI lesions is similar to IFN-*β*, however, GA has somewhat limited effect on disability progression (Rudick et al. 1997; La Mantia et al. 2010). The side effect profile of GA is, however, more favorable and includes local injection site reactions, post injection reaction (flushing, chest tightness, palpitation, and dyspnea within minutes of injection with spontaneous resolution) and rare lipoatrophy with prolonged use.

## Natalizumab

Natalizumab is a humanized monoclonal antibody that binds *α*4*β*1-integrin on lymphocytes blocking their interaction with VCAM-1 on endothelial cells thereby preventing the transmigration of lymphocytes across the BBB (Ransohoff 2007). Its superior efficacy has been demonstrated in two phase 3 studies with a robust effect on relapse rate reduction and disability progression (Miller et al. 2003; Polman et al. 2006). The major safety concern with natalizumab is progressive multifocal leukoencephalopathy (PML), a serious potentially fatal opportunistic brain infection caused by reactivation of JC virus (Yousry et al. 2006). As of December 2014, 514 cases of PML have been reported worldwide with postmarketing use of natalizumab (TYSABRI [natalizumab] 2014). The overall risk of PML in MS patients with natalizumab use is 3.78 per 1000 with a much higher risk (13/1000) among patients with prolonged duration of therapy (≥24 months), history of prior immunosuppressive therapy, and positive JC virus antibody status (Bloomgren et al. 2012; Information M, 2014). Due to the risk of PML, natalizumab now has a more limited use as a second line drug in patients with breakthrough disease or intolerable side effects with first line therapies.

## Mitoxantrone

Mitoxantrone is a synthetic anthracendione antineoplastic agent; its immunomodulatory effects include suppression of T and B lymphocytes and macrophage proliferation. Mitoxantrone is indicated for reducing disability and relapse frequency in patients with worsening relapsing remitting and secondary-progressive MS, however, its use has been limited due to risk of dose-related cardiotoxicity and treatment-related leukemia (Fox 2006).

## Oral Therapies

Three new oral medications have recently become available for treatment of relapsing MS: fingolimod, teriflunomide, and dimethylfumarate. The efficacy of these medications has been established in several phase 3 studies with comparable or somewhat better effect (compared to some injectable therapies) on relapse rate reduction, MRI lesions, and disability progression.

*Fingolimod* is a sphingosine-1-phosphate receptor (S1P1) modulator and was the first oral drug approved for treatment of MS. By binding to S1P1 receptor on the T cells, it prevents emigration of activated T cells from lymph nodes thereby limiting their entry into the CNS (Chiba et al. 1998; Pelletier and Hafler 2012). The potential side effects of fingolimod include first dose bradycardia, atrioventricular block, herpes virus infection, macular edema, elevated blood pressure, and a reported cases of PML (Cohen et al. 2010; Kappos et al. 2010; Samson 2013; Calic et al. 2015).

There have been a total of three reported cases of PML associated with use of fingolimod, two of these occurred in context of prior immunosuppressive therapy, the third most recent case, however, was reported in a patient with no prior immunosuppressive therapy after more than 4 years of fingolimod use (Calic et al. 2015; Case of PML reported in patient treated with Gilenya, 2015). A single case of sudden suspected cardiac death within 24 h of taking first dose of fingolimod was reported in December 2011 (Lindsey et al. 2012). Even though a direct association with the medication could not be established, the US Food and Drug Administration and European regulatory agency released new monitoring guidelines for first dose monitoring and the drug is now contraindicated in patients with history of cardiac disease or stroke and patients on antiarrhythmic medications.

*Teriflunomide* is an active metabolite of leflunomide (a drug used to treat rheumatoid arthritis) and is an inhibitor of enzyme dihyrdro-orotate dehydrogenase (DHODH) which interferes with denovo synthesis of pyrimidine in rapidly dividing cells (Oh and O’Connor 2013). Its anti-inflammatory effect in MS is believed to be mediated by reducing the activity of proliferating T and B lymphocytes. Teriflunomide does not affect the resting or slowly dividing hematopoietic cells which use the alternate DHODH independent “salvage pathway” for pyrimidine synthesis, therefore, preserving the basic homeostatic functions of these cells and immune surveillance (Oh and O’Connor 2013). Leflunomide is converted almost entirely into teriflunomide after absorption and taken at the recommended doses, both drugs result in similar plasma concentration of teriflunomide (AUBAGIO® (teriflunomide) Prescibing information. 2014). The short-term side effects of teriflunomide are relatively mild and include hair loss, headache, diarrhea, and elevated liver enzymes (O’Connor et al. 2011). Reduction in lymphocyte and neutrophil counts, elevated blood pressure, and a single case of latent tuberculosis are some of the other side effects reported (O’Connor et al. 2011; Confavreux et al. 2014). The potential teratogenecity of teriflunomide remains a major concern although several pregnancies reported during its clinical trial did not have any adverse outcome. Nevertheless, strict contraception is recommended to avoid pregnancy and a rapid elimination process is undertaken in women who become pregnant while taking teriflunomide as the drug can remain in the systems for 8 months to 2 years (Confavreux et al. 2014).

*Dimethylfumarate (DMF) or BG12* is the latest oral therapy to be approved for treatment of MS. Related to fumaric acid ester which has been used for treatment of psoriasis in Germany since 1990s; BG12 is as an enteric coated formulation of DMF with improved GI tolerability. It is hydrolyzed to monomethyl fumarate soon after oral absorption. The mechanism of action of DMF involves inhibition of proinflammatory pathways via activation of nuclear factor erythroid 2-related factor 2 (Nrf-2) antioxidant response pathway (Linker et al. 2011). The most common side effect with DMF include nausea, diarrhea, abdominal pain which can be minimized by taking the medication with food and flushing which can be reduced by aspirin (Gold et al. 2012; Fox et al. 2014). Lymphopenia may occur although no increased infection risk was observed in phase 3 studies (Fox et al. 2012; Gold et al. 2012). There have been a few cases of PML reported with use of fumaric acid ester formulations in patients with psoriasis with pronounced prolonged lymphopenia being the major risk factor (Ermis et al. 2013; van Oosten et al. 2013; Sweetser et al. 2013). There has been a recent report of a fatal case of PML in a patient with multiple sclerosis treated with dimethyl fumarate (FDA Drug Safety Communication, 2014). In response to these cases of PML, JC virus antibody testing and close monitoring for lymphopenia has been suggested in patients initiating DMF therapy to improve surveillance.

**Figure 3:**
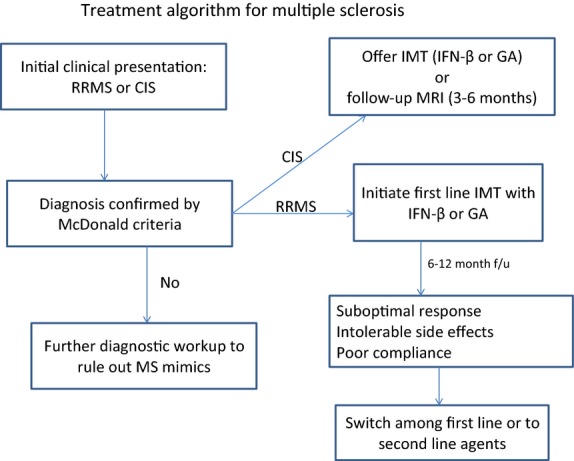
Treatment approach to patients with RRMS and CIS.

The oral medications are currently considered second line due to a greater risk of serious side effects making their safety profile less favorable in patients with early and mild disease. The main safety concern with oral medications is the potential for prolonged immunosuppression increasing the risk of serious infections and also malignancies due to altered immune surveillance. A simplified treatment algorithm for patients with RRMS and CIS is outlined in Figure3.

In summary, there has been significant progress in the field of MS with better understanding of immunopathogenesis, wider availability and use of MRI, and availability of disease modifying therapies. There are currently a wide range of therapeutic options to treat MS including the recently approved oral drugs. However, balancing the safety and efficacy of these drugs remains a challenge due to serious side effects associated with more effective therapies. Given the heterogeneity of MS, personalized treatment by recognizing specific genetic markers in individual patients has been proposed. Also, there is an urgent need for novel therapeutic agents that can prevent or minimize the neuronal and/or axonal degeneration occurring in patients with progressive MS.

## Conflict of Interest

None declared.
